# Evidence for Exercise Therapy in Elderly Patients with Rheumatoid Arthritis

**DOI:** 10.14789/ejmj.JMJ24-0039-R

**Published:** 2025-03-12

**Authors:** MASASHI AOYAGI, HIDERU ITO, RYOTA KURATSUBO, YUKI SHIOTA, TAKAYUKI KOMATSU, YUJI TAKAZAWA

**Affiliations:** 1 Juntendo Administration for Sports, Health and Medical Sciences, Juntendo University, Tokyo, Japan; 1 Juntendo Administration for Sports, Health and Medical Sciences, Juntendo University, Tokyo, Japan; 2 Graduate School of Medicine, Juntendo University, Tokyo, Japan; 2 Graduate School of Medicine, Juntendo University, Tokyo, Japan; 3 Sports Medicine, School of Health and Sports Science, Graduate School of Health and Sports Science, Juntendo University, Chiba, Japan; 3 Sports Medicine, School of Health and Sports Science, Graduate School of Health and Sports Science, Juntendo University, Chiba, Japan; 4 Department of Sports Medicine, Faculty of Medicine, Juntendo University, Tokyo, Japan; 4 Department of Sports Medicine, Faculty of Medicine, Juntendo University, Tokyo, Japan

**Keywords:** aging, physical fitness, evidence-based research

## Abstract

**Objectives:**

The number of elderly patients with rheumatoid arthritis (RA) is increasing due to the extension of life expectancy and advances in pharmacotherapy. Although exercise therapy has shown to be effective in patients with RA, there is a lack of evidence specifically focusing solely on elderly patients. We aimed to review the evidence for the effects of exercise therapy on elderly patients with RA.

**Design:**

Systematic review.

**Methods:**

An electronic search was conducted on June 30th, 2023, in five online databases. Controlled clinical trials investigating the effects of exercise therapy on elderly patients with RA were included. No restrictions were placed on the exercise therapy or outcomes to examine the effects of exercise therapy broadly. The risk of bias in the included studies was assessed using the revised Cochrane risk-of-bias tool for randomized trials and the risk of bias in non-randomized studies of interventions.

**Results:**

Out of the 4,177 articles identified, only three studies were included. All three studies were judged to have a high or serious risk of bias. The exercise therapies in the included studies were Arthritis self-management programs, Tai Chi, and grip-strengthening exercise. Arthritis self-management programs and Tai Chi showed the positive effects, whereas grip-strengthening exercise reported no effectiveness.

**Conclusions:**

In a few controlled clinical trials, the effects of exercise therapy on elderly patients with RA have been investigated, suggesting that the evidence on these effects is very limited. Considering that the society will continue to age, further studies on this topic are required.

## Introduction

Rheumatoid arthritis (RA) is a chronic systemic inflammatory disease caused by autoimmune abnormalities. A typical symptom is inflammation of the synovial membranes of the joints. As arthritis progresses, joints become deformed and function declines, leading to impaired activities of daily living (ADLs) and quality of life (QOL)^1)^. In general population, the prevalence of RA is approximately 1%^2-4)^. However, due to extended life expectancy and advances in pharmacotherapy, the total number of patients is on the rise and has reportedly increased by 7.4% between 1990 and 2017^5)^. In particular, as society ages, the number of elderly patients who develop RA at an older age is increasing^6-8)^. In Finland, the peak age at RA onset has increased from 50.2 to 57.8 years over a 15-year period^6, 7)^. In Japan, where the population is aging rapidly, the peak age at RA onset was prolonged into the 60s in 2012-2013, while it was the 50s in 2002-2003^8)^. In addition, owing to the recent advent of biological agents and molecularly targeted antirheumatic drugs, the therapeutic goals for RA have also shifted from reducing clinical symptoms to improving and maintaining patients’ physical function and QOL by controlling disease activity and minimizing joint destruction. Thus, medical treatments that specifically focus on physical function and QOL in elderly patients with RA is required.

There are noticeable physical function issues in elderly patients with RA. They have low muscle mass and high body fat compared to those of healthy controls, which can lead to sarcopenia^9)^. In addition, when muscle mass decreases, the risk of falls and fractures could increase. Additionally, these patients are at a heightened risk for frailty, a condition characterized by diminished physiological function and increased vulnerability to adverse health outcomes^10)^. Frailty in older adults with RA is exacerbated by functional disability, reduced physical activity due to pain and joint deformities, and comorbidities such as cardiovascular disease. As RA progresses, these factors can accelerate declines in physical function and QOL. Therefore, for elderly patients with RA, exercise might be important to maintain physical function. However, previous studies reported that the level of physical activity in elderly patients with RA is lower than that in healthy elderly people^11, 12)^, with only 13.8 % of patients exercising at least three times per week^13)^. This is partly because, although these patients understand the importance of exercise, they do not know what type of exercise will not adversely affect their joints^14, 15)^.

Previous studies on examination of the long-term effects of exercise therapy in patients with RA reported no adverse effects on disease activity or joint function^16)^. Previous systematic reviews have also reported improvement in physical function and increase in muscle mass as benefits of exercise therapy^17, 18)^. However, these studies were conducted on both younger and older patients with RA. As elderly patients with RA may have comorbidities such as pulmonary disease and malignancy, in addition to the limitation in range of motion and muscle weakness, conducting the same intensity of exercise therapy in elderly patients as that conducted in younger patients may be difficult. Therefore, in this systematic review, we aimed to examine the evidence for the effects of exercise therapy on elderly patients with RA.

## Methodology

### Protocol

This systematic review was conducted according to the Preferred Reporting Items for Systematic Reviews and Meta-Analyses (PRISMA) guidelines^19)^.

### Eligibility criteria

#### Types of studies

Controlled clinical trials, including randomized (RCTs) and non-randomized controlled trials (non- RCTs), were included in this review. Articles written in English or Japanese were included. Studies without control groups, including single-arm trials, observational studies, and conference proceedings, were excluded.

#### Participants

In targeted clinical studies, patients with RA aged 50 years or older, and with a mean or median age of 60 years or older for the entire population were included. Patients with other types of arthritis, including osteoarthritis, were excluded.

#### Intervention

Interventions of interest were all types of exercise therapies that utilized exercise to maintain or improve symptoms and physical functions in patients with RA, such as aerobic, muscle strengthening, and underwater exercises.

#### Comparison

The control groups included non-physical interventions such as no treatment, education, pharmacotherapy, and information provision.

#### Outcome measures

To broadly examine the effects of exercise therapy, no restrictions were placed on the types of outcomes such as muscle strength, pain, disease activity, cardiopulmonary function, quality of life, ADL, biochemical data, or walking speed.

### Literature search

An electronic literature search was conducted across the following databases from their inception until June 30, 2023: PubMed, Cochrane Central Register of Controlled Trials (CENTRAL), Web of Science, Cumulative Index to Nursing and Allied Health (CINAHL), and Ichushi-Web (a Japanese database). Web of Science includes studies dating back to 1900, ensuring comprehensive coverage. The comprehensive search strategy was established in consultation with a university librarian to identify relevant studies in both English and Japanese. Keywords such as “Arthritis”, “Rheumatoid”, “Exercise”, “Training”, “Rehabilitation”, and Clinical trial” were used (Supplementary Table 1). Two reviewers independently performed literature searches.

### Study selection

**Figure 1:**
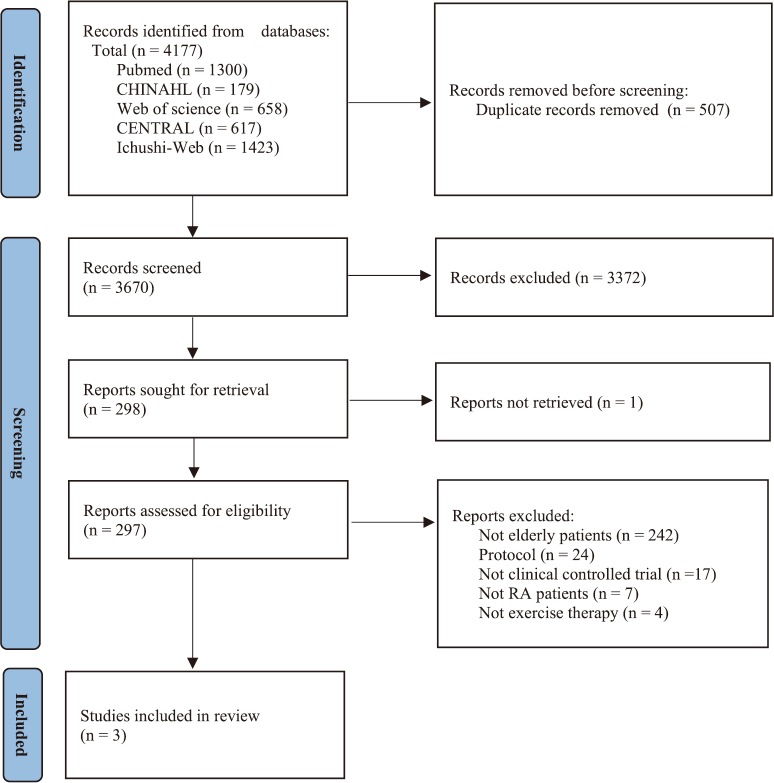
The process of study selection RA: rheumatoid arthritis

Two reviewers independently screened and reviewed all articles for eligibility (Figure 1) according to the criteria. Any disagreements between the reviewers were resolved through discussion with reference to the criteria. In cases where an agreement was not obtained, a third reviewer was brought in to discuss and reach a consensus.

### Data extraction

Data were extracted from studies that met the eligibility criteria by one reviewer in a standardized form, and the accuracy of the data was checked by another reviewer. The extracted data included study design, participants, content of the intervention, dropouts, outcomes, and results. To ascertain adverse reactions to exercise therapy, the number of participants who dropped out and the reasons for dropping out were recorded. The number of participants was used for statistical analysis. Missing data were excluded after contacting the corresponding authors.

### Quality assessment of studies

The risk of bias in the included studies was assessed using the revised Cochrane risk-of-bias tool for randomized trials (RoB 2) for RCTs, and the risk of bias in non-randomized studies of interventions (ROBINS-1) for non-RCTs^20, 21)^. Using RoB 2, the overall risk of bias was classified as low risk of bias, some concerns, and high risk of bias, whereas using ROBINS-1, the risk was classified as low, moderate, serious, and critical risk of bias, and no information. One reviewer assessed the risk of bias in each included study, and another reviewer checked for accuracy. In cases of disagreement, the results agreed upon by both reviewers were adopted after discussion.

### Data analysis

The standard mean difference (SMD) with a 95% confidence interval (CI) was calculated for each reported outcome to evaluate the effects of exercise therapy. Calculations were performed using Review Manager version 5.4.1, developed by The Cochrane Collaboration (revman.cochrane.org).

## Results

### Study selection

Figure 1 shows the process of selecting the studies included in this review. A total of 4,177 articles were identified through the literature search. After excluding 508 duplicates, 3,670 articles were screened for titles and abstracts. After screening, 267 articles were examined for eligibility by reviewing the full text. Three studies were included in the systematic review. Due to the small number of included studies, a meta-analysis was not performed.

### Characteristics of the included studies

Data extracted from the included papers are summarized in Table 1; one study was an RCT^22)^, and two were non-RCTs^23, 24)^. In all three studies, the participants were women, and their ages ranged from 50 to 87 years. The disease activity of RA was stable in two studies^23, 24)^, whereas one study did not report it^22)^. None of the studies reported the stages of RA. The interventions included arthritis self-management programs, which consisted of education and exercises, including stretching, endurance, and light resistance exercises^22)^, Tai Chi^24)^, and grip-strengthening exercises^23)^. In terms of the duration and frequency of intervention, the exercises in the arthritis self-management program were conducted once a week for 30 minutes for six weeks^22)^, Tai Chi was performed once a week for 60 minutes for three months^24)^, and grip-strengthening exercises were conducted every day for eight weeks^23)^, suggesting significant variations between studies. Outcomes used in included studies were self-efficacy function and pain scale^22)^, color fraction value at the wrist^23)^, and measurements related to atherosclerosis and cardiovascular disease^24)^. No objective outcomes related to physical function and QOL were used.

The arthritis self-management program and Tai Chi were conducted in a group setting^22, 24)^, whereas grip-strengthening exercises were conducted individually^23)^. Dropouts during the intervention period included three of 40 participants in the intervention group and one of 40 participants in the control group in the arthritis self-management program due to loss to follow-up^22)^. Overall, 13 of 27 participants in the control group in the Tai Chi program were lost to follow-up due to the loss of interest in the study^24)^, and six of 24 participants in the grip- strengthening exercise intervention group were lost to follow-up due to pain or worsening of disease activity caused by the exercises^23)^.

**Table 1:** Characteristics of the included studies

Article	Study design	Participants	Intervention and Control	Dropout	Outcomes	Results
Anvar et al. (2018)^22)^	RCT	80 patients (0 men and 80 women) Age Intervention group: 69 y (60-87) Control group: Not mentioned Disease duration Intervention group: Not mentioned Control group: Not mentioned Disease activity: Not mentioned	Intervention: Arthritis self-management program Frequency: 1/week Time: 30 minutes Duration: 6 weeks Control: Booklet about educational content	Intervention: 3 patients (unknown reason) Control: 1 patient (unknown reason)	Self-efficacy function scale Self-efficacy pain scale	Self-efficacy function scale: Significantly improved compared to that in the control group Self-efficacy pain scale: Significantly improved compared to that in the control group
Ellegaard et al. (2013)^23)^	non-RCT	42 patients (0 men and 42 women) Age Intervention group: 60 y (53-70) Control group: 62 y (53-71) Disease duration Intervention group: 8 y Control group: 10 y Disease activity: Stable	Intervention: Grip-strengthening exercise Frequency: 10-15 repetitions/day Time: Not mentioned Duration: 8 weeks Control: Medication	Intervention: 6 patients (Worsening pain or flaring up disease activity) Control: 0 patient	Color fraction value (at the wrist)	Color fraction value: No difference
Shin et al. (2015)^24)^	non-RCT	56 patients (0 men and 56 women) Age Intervention group: 64 y (50 and above) Control group: 60.9 y (50 and above) Disease duration Intervention group: 10.3 y Control group: 15.3 y Disease activity: Stable	Intervention: Tai Chi Frequency: 1/week Time: 60 minutes Duration: 3 months Control: Provide information about lifestyle modification and regular exercises	Intervention: 0 patient Control: 13 patients (lost interest in the study)	Atherosclerotic measurement: cIMT FMD baPWV CVD risks: BP Heart rate Total cholesterol RA characteristics: DAS28-ESR RAPID3 HAQ	Atherosclerotic measurement: cIMT: No difference FMD: Significantly increased compared to that in the control group baPWV: Significantly decreased compared to that in the control group CVD risks: BP: No difference Heart rate: No difference Total cholesterol: Significantly decreased compared to that in the control group RA characteristics: DAS28-ESR: No difference RAPID3: No difference HAQ: No difference

baPWV: brachial-ankle pulse wave velocity; BP: blood pressure; cIMT: carotid intima-media thickness; CVD: cardiovascular disease; DAS28-ESR: disease activity score 28-erythrocyte sedimentation rate; FMD: flow-mediated dilatation; HAQ: Health Assessment Questionnaire; RA: rheumatoid arthritis; RAPID3: routine assessment of patient index data 3; RCT: randomized controlled trial; y: years

### Effects of exercise therapy

In the arthritis self-management program, the intervention group demonstrated significant improvements in self-efficacy function scores (SMD: 7.53, 95% CI: 6.22 to 8.84) and pain scores (SMD: 10.27, 95% CI: 8.53 to 12.00) compared with those in the control group (Odds ratio was unknown)^22)^. For the Tai Chi therapy, the intervention group showed significant improvements in flow-mediated dilation, which indicated vascular endothelial function, (SMD: 1.11, 95% CI: 0.42 to 1.79) and brachial-ankle pulse wave velocity, which indicated arterial stiffness, (SMD: -0.81, 95% CI: -1.47 to -0.14) compared with those in the control group^24)^. In the grip- strengthening exercises group, no significant differences were observed in color fraction value at the wrist, which indicated inflammation-related findings (SMD: -0.67, 95% CI: -1.34 to 0.00)^23)^.

### Quality assessment of studies

Tables 2 and 3 present the results of the risk of bias assessment. One study was judged to have a “high risk of bias^22)^,” and two studies were judged to have “serious of risk of bias^23, 24)^.”

**Table 2:** Risk of bias of the included study estimated using the revised Cochrane risk-of-bias tool for randomized trials (RoB2)

Article	D1	D2	D3	D4	D5	Overall
Anvar et al. (2018)^22)^	Some concerns	Some concerns	Low	High	Some concerns	High risk of bias

D1: bias arising from the randomization process; D2: bias due to deviations from the intended intervention; D3: bias due to missing outcome data; D4: bias in the measurement of the outcome; D5: bias in the selection of the reported result.

**Table 3:** Risk of bias of the included studies estimated using the risk of bias in non-randomized studies of interventions (ROBINS-1)

Article	D1	D2	D3	D4	D5	D6	D7	Overall
Ellegaard et al. (2013)^23)^	Low	Serious	Low	Serious	Serious	Low	Moderate	Serious risk of bias
Shin et al. (2015)^24)^	Low	Low	Low	Serious	Serious	Low	Moderate	Serious risk of bias

D1: bias due to confounding data; D2: bias due to selection of participants; D3: bias in classification of interventions; D4: bias due to deviations from intended interventions; D5: bias due to missing data; D6: bias in the measurement of the outcomes; D7: bias in the selection of the reported results.

## Discussion

In this systematic review, we examined the effects of exercise therapy on elderly patients with RA. Although 4,177 articles were screened, only three studies met the inclusion criteria^22-24)^. Of the three studies, two studies where the patients underwent the arthritis self-management program and Tai Chi therapy showed positive effects on self-reported function, pain, vascular endothelial function, and arterial stiffness^22, 24)^, although the other study where patients performed grip-strengthening exercises did not show any changes in the inflammation-related findings^23)^. This suggests that arthritis self-management programs and Tai Chi may be beneficial for elderly patients with RA. However, considering the small number of included studies, which involved only women patients, substantial heterogeneity in the intervention programs between the studies, and high or serious risk of bias in all studies, the current evidence for the positive effects of exercise therapy in elderly patients with RA is limited.

Maintaining physical function is important for sustaining the QOL of elderly patients. In particular, elderly patients with RA have a higher incidence of sarcopenia than that in elderly individuals of the same age without RA^25)^, suggesting that elderly patients with RA are prone to the loss of physical function. Although exercise therapy is effective in maintaining physical function, many patients with RA hesitate to exercise because of concerns that it may worsen their arthritis^14, 15)^. No adverse reactions were reported in the included studies where the arthritis self-management program or Tai Chi were conducted^22, 24)^. On the other hand, grip- strengthening exercises exacerbated pain or disease activity in six patients (25%)^23)^; however, a previous study reported that hand exercises, including grip-strengthening exercises, did not cause adverse events in patients with RA^26)^. The authors reported that disease activity in those who dropped out at the baseline assessment was higher than that in those who completed the intervention^23)^. Thus, to safely implement exercise therapy, the intensity, location, and frequency of exercise should be determined based on disease activity.

Previous systematic reviews have reported positive effects of exercise therapy on aerobic capacity, walking speed, hand function, fatigue, and muscle strength in patients with RA^17, 18)^. However, these reviews did not analyze younger and older patients with RA separately. Because physical function tends to decline with advancing age, exercise therapy that places the same load on both groups of patients may be more demanding for older patients. Therefore, establishment of evidence of the effects of exercise therapy is required, specifically in elderly patients with RA. As the current systematic review revealed that only a few studies have been conducted on such patients, and all of them contained methodological issues, further studies, especially high-quality RCTs, are warranted to provide evidence-based exercise therapy.

This systematic review highlights some of the difficulties in investigating the effects of exercise therapy in elderly patients with RA. Particularly, standardizing the physical functions and backgrounds of participants with RA is a major challenge. Physical function in the elderly varies widely among individuals depending on several factors such as physical activity level and medical conditions. In addition, in patients with RA, physical function can vary depending on the duration of the disease and whether disease activity is controlled. Therefore, when the same exercise therapy was implemented for different individuals, differences were observed in the amount of load applied to each participant, which could affect the effects of the therapy. To address this challenge, large studies, in which patients are grouped according to their physical function and disease activity, are required.

This study had several limitations. First, the number of included studies was limited. Two studies examining the effects of exercise therapy on elderly patients with RA were excluded because they were single-arm studies and provided home-based exercises to the control group^27, 28)^. These studies investigated high-intensity interval walk training and aerobic and resistance exercises, reporting potential benefits for elderly patients with RA^27, 28)^. However, even if these studies were included, the conclusion would still be that only a few studies have examined the effects of exercise therapy in this population. Second, the content and duration of exercise therapy and outcomes varied considerably across the included studies. This is probably because RA is a systemic disease that affects a wide range of physical functions, making standardization of exercise therapy and outcomes difficult. Third, we included studies that employed patients over 50 years of age with RA. Although the elderly were over 60 or 65 years of age^29)^, we believe that this difference had little impact on the current results, as the mean or median age of the entire population in the included studies was 60 years or older. Fourth, elderly patients with RA can be categorized into elderly-onset RA (disease onset after 60 years of age) and non-elderly-onset RA, with each group potentially responding differently to exercise therapy due to differences in disease progression, symptoms, and physical function^30, 31)^. However, none of the included studies provided data on disease onset, making it impossible to analyze these subgroups. Similarly, the absence of information on the RA disease stage, which influences physical function and exercise tolerance, limits the interpretation of results. Fifths, the participants in the included studies were only women. As physical function, including muscle strength and muscle mass, may be different between sexes, studies with male patients may be needed. Finally, although exercise could be important in maintaining physical function and QOL in elderly patients with RA, as those patients are at risk of developing sarcopenia^9)^, no objective outcomes related to physical function and QOL were used in the included studies. Evaluating such outcomes is crucial to ensuring the clinical relevance of findings. Further studies should adopt standardized protocols, measure subjective and objective outcomes of physical function (for example, muscle strength and walking speed) and QOL, and stratify patients based on onset type, disease stage, and activity level. This approach will address heterogeneity in patient characteristics, enhance generalizability, and provide stronger evidence for the benefits of exercise therapy.

## Conclusions

Only a small number of controlled clinical trials have been conducted to investigate the effects of exercise therapy in elderly patients with RA. Additionally, no studies have been conducted using a robust methodology. Therefore, the current evidence is limited. Considering the social background, in which the number of elderly patients with RA is expected to increase in the future, high-quality research on the current topic is desired.

## Funding

The authors received no financial support for this research.

## Author contributions

MA and HI performed the literature search and data synthesis. RK, YS, TK, and YT contributed to the study design, and data analysis. All authors reviewed and approved the final manuscript.

## Conflicts of interest statement

The authors declare that there are no conflicts of interest.

## Supplementary Material

**Supplementary Table 1:** Search strategy for electronic databases

PubMed	#1	(“Arthritis, Rheumatoid”[MeSH] or arthritis[TIAB])
	#2	(“Exercise”[MeSH] or “Exercise Therapy”[MeSH] or “Physical Education and Training”[MeSH] or “Physical Fitness”[MeSH] or “Resistance Training”[MeSH] or “Rehabilitation”[MeSH] or “Physical Therapy Modalities” [MeSH] or “Occupational Therapy”[MeSH] or “Physical Fitness”[MeSH]) or exercise*[TIAB] or “motion therapy*”[TIAB] or “physical education*”[TIAB] or training[TIAB] or “water therapy*”[TIAB])
	#3	(“clinical trial”[Publication Type] or “clinical trials as topic”[MeSH Terms])
	#4	#1 and #2 and #3
CENTRAL	#1	(MeSH descriptor: [Arthritis, Rheumatoid] explode all trees)
	#2	(MeSH descriptor: [Exercise] explode all trees) or (MeSH descriptor: [Exercise Therapy] explode all trees) or (MeSH descriptor: [Physical Fitness] explode all trees) or (MeSH descriptor: [Resistance Training] explode all trees) or (MeSH descriptor: [Rehabilitation] explode all trees) or (MeSH descriptor: [Physical Therapy Modalities] explode all trees) or (MeSH descriptor: [Occupational Therapy] explode all trees) or (MeSH descriptor: [Physical Education and Training] explode all trees) or (exercise*:ab,ti) or (physical education*:ti,ab) or (training:ti,ab))
	#3	#1 and #2
Web of Science	#1	(“Rheumatoid arthritis” or “arthritis”)
	#2	(“Exercise” or “Exercise Therapy” or “Physical Education and Training” or “Physical Fitness” or “Resistance Training” or “Rehabilitation” or “Physical Therapy Modalities” or “Occupational Therapy” or “Physical Fitness”) or (“exercise*” or “motion therapy*” or “physical education*” or “training” or “water therapy*” or “physical therapy*”)
	#3	(“clinical trial” or “clinical trials as topic”)
	#4	#1 and #2 and #3
CHINAHL	#1	(MH “Arthritis, Rheumatoid”) or (MH “Arthritis”)
	#2	(MH “Exercise”) or (MH “Therapeutic Exercise”) or (MH “Physical Education and Training”) or (MH “Physical Fitness”) or (MH “Resistance Training”) or (MH “Rehabilitation”) or (MH “Combined Modality Therapy”) or (MH “Occupational Therapy”) or (MH “Physical Fitness”) or (MH “Exercise”) or (MH “Motion Therapy, Continuous Passive”) or (MH “Education, Physical Therapy”) or (“training” or “water therapy*” or “Physical therapy*”)
	#3	(MH “Clinical Trial Registry”) or (MH “Clinical Trials”)
	#4	#1 and #2 and #3
Ichushi-Web	#1	(関節リウマチ/TH or 関節リウマチ/TA)
	#2	((運動療法/TH or トレーニング/TH or 水治療法/TH or リハビリテーション/TH) or (運動療法/TA or トレーニング/TA or 水治療法/TA or リハビリテーション/TA))
	#3	(PT=原著論文)
	#4	#1 and #2 and #3

MeSH: medical subject headings
